# Ammonia and Ammonium Exposure of Basil (*Ocimum basilicum* L.) Growing in an Organically Fertilized Peat Substrate and Strategies to Mitigate Related Harmful Impacts on Plant Growth

**DOI:** 10.3389/fpls.2019.01696

**Published:** 2020-02-28

**Authors:** Christian Frerichs, Diemo Daum, Andreas Siegfried Pacholski

**Affiliations:** ^1^ Faculty of Agricultural Sciences and Landscape Architecture, Osnabrueck University of Applied Sciences, Osnabrueck, Germany; ^2^ EuroChem Agro, Mannheim, Germany

**Keywords:** pot grown basil (*Ocimum basilicum* L.), organic cultivation, liquid amino acid fertilizer, ammonia and ammonium toxicity, substrate pH, nitrification, nitrate, mature compost

## Abstract

Organic pot-based production of basil (*Ocimum basilicum* L.) often has lower biomass yield than conventional cultivation. Previous investigations indicate that this growth impairment is related to high ammonium (NH_4_^+^) concentrations in the growing media released by the mineralization of organic nitrogen (N) fertilizers. However, as a result of this ammonification process substrate pH may also increase. Under neutral to alkaline conditions NH_4_^+^ is converted to ammonia (NH_3_), which is known to be phytotoxic even at low concentrations. Therefore, we investigated the impact of both ammonical N species on basil grown in a peat substrate. In total, three fertilization pot experiments were conducted in a greenhouse in order to compare the effect of different organic base dressings [250 and 750 mg N (L substrate)^-1^ mainly supplied by a liquid amino acid fertilizer (AAF)] and two initial substrate pH levels (5.5 and 6.5). In two treatments, 5% (v/v) mature compost was mixed into the peat 1 day and 12–days before the substrate was used for sowing, respectively. The aim of this procedure was to stimulate nitrification in this way to reduce ammonical N concentration. Ammonia concentration in the aerial plant surrounding environment was measured by using NH_3_ detector tubes in combination with an open-top chamber method. The results showed that the growth of basil (number of plants, fresh matter yield, plant height) was significantly inhibited in the second and third week of cultivation by rising NH_3_ and NH_4_^+^ exposure, as well as by a substrate pH ≥ 7.0. These adverse effects were reduced by lowering the organic base dressing rate and adjusting the initial substrate pH to 5.5. Furthermore, the addition of mature compost to peat in combination with a 12-day storage was proven to be effective for promoting nitrification in the organically fertilized substrate. As a result, plant growth was improved by both lower NH_3_ and NH_4_^+^ exposure as well as a faster supply of nitrate (NO_3_^-^) as an additional N source. Using this approach, it was possible to feed organically fertilized basil right from the seedling stage with a NO_3_^-^-N/NH_4_^+^-N-balanced and later on providing a predominant NO_3_^-^-N supply.

## Introduction

Along with the trend towards natural flavoring of food as well as a health-conscious nutrition, demand for potted herbs in many European countries is raising (CBI, 2016a). These plants grow in an organic substrate until use by the consumer, and thus provide optimal freshness as well as a longer shelf life than fresh-cut produce. Potted herbs make up about two-thirds of the total fresh herbs sales in Germany (Statista, 2018). Within this market segment basil (*Ocimum basilicum* L.) accounts for 50% of the turnover (CBI, 2016b). Pot-grown basil in greenhouses is produced throughout the year and about 26% of traded goods complies with the European regulations for organic farming (EC 834/2007) (AMI, 2018).

Compared to conventional cultivation, organic cultivation of basil often leads to lower yield and quality. As early as 1 to 2 weeks after germination, cotyledons may become chlorotic and necrotic in organic production systems. During the winter period, these disorders are frequently accompanied by fungal diseases such as *Botrytis* (Frerichs et al., 2017). A previous investigation (Frerichs et al., 2019) showed it was possible to induce similar symptoms when basil was fed with NH_4_^+^ as sole N source, stabilized by a nitrification inhibitor. Even higher damage appeared following organic N fertilization. In this treatment, plants were exposed to particularly high NH_4_^+^ concentrations at the beginning of the cultivation period, because the added organic fertilizer was rapidly mineralized. In contrast, a NO_3_^-^ or balanced mineral N supply with NH_4_^+^ and NO_3_^-^ led to a significantly higher biomass production without any damage to plants. Similar effects of the N form on growth of basil were reported by Kiferle et al. (2013). Overall, these results indicate that basil responds sensitively to high concentration of NH_4_^+^, as is well known for many plant species (Britto and Kronzucker, 2002).

Consequently, we hypothesized that growth impairments of basil in organic production are related to a temporary excessive NH_4_^+^ supply. Various mechanisms may contribute to this phenomenon. Beside toxicity of NH_4_^+^ itself (Liu and von Wirén, 2017) indirect effects such as NH_4_^+^-induced pH changes in the growing medium must also be taken into account. Roots release protons (H^+^) when absorbing NH_4_^+^ to maintain a stable intracellular pH (Van Beusichem et al., 1988). Furthermore, the oxidation of one mole NH_4_^+^ to NO_3_^-^ *via* nitrite (NO_2_^-^) by nitrifying bacteria generates two moles of H^+^ (Sahrawat, 2008). Thus, fertilization with NH_4_^+^ promotes the acidification of the rhizosphere (Wiesler, 1997). However, following organic N fertilization, a transient pH increase by the ammonification process can occur before the resulting NH_4_^+^ is converted by nitrification (Vetanovetz and Peterson, 1990; Niemiera et al., 2014). With rising pH, the NH_4_^+^/NH_3_ equilibrium shifts towards NH_3_. Significant NH_3_ formation starts from a pH of about 7.0 (Lægreid et al., 1999). Neutral to slightly alkaline conditions may arise in growing media containing substantial proportions of peat substitutes like composts and wood fibers. These constituents, common in organic production of pot plants, are relatively rich in calcium carbonate (CaCO_3_) or have a weak buffer capacity against alkaline shifts (Neumaier and Meinken, 2015). High temperatures, typical in greenhouse cultivation, further favor the formation of NH_3_ (Lægreid et al., 1999). Ammonia is known to be phytotoxic even at low concentrations, both in dissolved form in the rhizosphere and in gaseous form in the plant canopy environment (Schenk and Wehrmann, 1979; Krupa, 2003).

To separate effects of NH_3_ and NH_4_^+^ on growth of organically fertilized basil, it is necessary to monitor both compounds over the cultivation period. Diffusion and drift processes between closely adjoining plots can hamper the detection of the exact NH_3_ exposure within the single plots (Gericke et al., 2011). Several approaches were developed to overcome these problems (Yang et al., 2018). A quick and easily implementable method to quantify the NH_3_ volatilization even in small-scale crop units is the “Draeger-Tube-Method”, which is based on the use of NH_3_ detector tubes in combination with a dynamic chamber (Pacholski et al., 2006; Pacholski, 2016). If only the NH_3_ concentration in the ambient air is relevant, as in the present study, open-top chambers can also be used (Chen et al., 2012).

Given the current state of knowledge, we hypothesized that the pH of the growing medium and the dosage of the organic N base dressing are crucial factors affecting the exposure of pot-grown herbs to NH_3_ and NH_4_^+^. An optimal initial substrate pH range is expected between 5.5–6.0 to avoid both significant NH_3_ exposure under neutral to alkaline conditions (Lægreid et al., 1999) and a reduced activity of nitrifying bacteria under acid conditions (Vetanovetz and Peterson, 1990; Lang and Elliott, 1991). Concentration of ammonical N in ready-to-use growing media might be further reduced by a storage treatment for several weeks after mixing the organic fertilizer into the substrate. During this time, easily decomposable fertilizer compounds can be mineralized to a larger extent (Koller et al., 2004; Nair et al., 2011). To facilitate subsequent nitrification, the addition of mature compost might be useful as well (Delics et al., 2017). In this way, the substrate will be enriched with a well-established population of nitrifying bacteria (Chroni et al., 2009; Zeng et al., 2012).

Thus, the objective of the present study was to investigate the NH_3_ and NH_4_^+^ exposure of pot-grown basil as affected by the amount of organic N applied with base dressing, the initial substrate pH as well as different storage treatments with and without addition of mature compost to a peat substrate. The following research questions were addressed: (a) To what extent is basil exposed to NH_3_ and NH_4_^+^ when cultivated in organically fertilized peat-based substrates? (b) What impact does this NH_3_ and NH_4_^+^ exposure have on the growth of basil? (c) Which practices may be suitable to minimize harmful NH_3_ and NH_4_^+^ exposure in organic basil production?

## Material and Methods

In total, three fertilization experiments were carried out from September 2016 to April 2017 in a greenhouse at the Osnabrueck University of Applied Sciences (**Table 1**). The first two trials focused on NH_3_ and NH_4_^+^ concentrations in response to different levels of organic N base dressing and substrate pH conditions, as well as the development of young basil plants under these conditions. The herbs were cultivated in autumn and winter seasons for a period of 4 and 5 weeks after sowing, respectively. The third trial was conducted to evaluate the efficacy of compost amendments to decrease phytotoxic NH_3_ and NH_4_^+^ exposure and high substrate pH under a high organic N base dressing level. Here, the plants were cultivated at the end of winter/beginning of spring for 8 weeks until they had reached a marketable size. In this way it was also examined whether plants are able to recover from high initial NH_3_ and NH_4_^+^ exposure in the seedling stage. Basil growth and yield were determined by measuring the plant height and by harvesting the shoot biomass at weekly intervals. Simultaneously, key substrate parameters (pH, NH_4_^+^, NO_3_^-^, NO_2_^-^, and total water-soluble salt concentration) were analyzed and the NH_3_ concentration in the canopy airspace was quantified with an open-top chamber approach as described below.

**Table 1:** Overview of the experimental setup in three fertilization trials.

Experiment no.	Treatment no.	Initial substrate pH	N supply [mg L (substrate)^-1^]	Compost addition and following substrate storage	
1 and 2	1 (control)	6.0	200 (BD with NO_3_^-^)	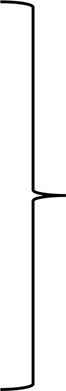	Without compost
	2	5.5	250 (BD with AAF)		
	3	5.5	750 (BD with AAF)		
	4	6.5	250 (BD with AAF)		
	5	6.5	750 (BD with AAF)		
3	6 (control)	6.5	200 (BD with NO_3_^-^) 400 (TD with NO_3_^-^)			
	7	6.5	750 (BD with AAF) 0 (TD with AAF)			
	8	6.5	750 (BD with AAF) 375 (TD with AAF)	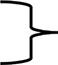	5% (v/v) compost was mixed into the substrate 12 days before sowing
	9	6.5	750 (BD with AAF) 250 (TD with AAF)	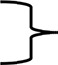	5% (v/v) compost was mixed into the substrate 1 day before sowing

Treatments differed in initial substrate pH, N base dressing (BD), and N top dressing (TD) by using NO_3_^-^ (control) or an amino acid fertilizer (AAF) as main component in the N supply. In addition, in experiment 3, treatments with compost addition and varying substrate storage periods were included.

### Plant Material and Experimental Conditions

Basil (*Ocimum basilicum* L.) var. “Edwina” was cultivated in 12-cm pots (0.6 L) filled with a peat substrate. In each pot, 50 seeds were sown on the substrate surface and covered with a white fleece tissue. After 6 days, seeds began to germinate and the cover was removed. In the first 15 days, plants were watered by overhead irrigation. Hereafter, water and fertilizer solution were supplied by periodic flooding of the pot saucer. For irrigation and fertigation deionized water was used. Air temperature was adjusted to 16°C/18°C (night/day). Between 6 a.m. and 8 p.m. the natural irradiance was supplemented by high pressure sodium lamps (44.3 µmol m^-2^ s^-1^) when irradiance outside the greenhouse dropped below 370.4 µmol m^-2^ s^-1^.

All experiments were performed in a randomized complete block design with three replications, each replication consisting of 25–36 pots. Every treatment and replication were positioned on a greenhouse table at a distance of 0.15–0.25 m from each other. Once a week, two pots were taken from every plot according to a randomization plan. The samples were used to measure NH_3_ concentration in the aerial environment of basil as well as destructively analyze plant and substrate parameters. After taking out these pots for the examinations, the remaining pots were positioned once again in a homogeneous square grid within the plots. Therefore, crop density decreased from 42 to 25 pots m^-2^ between week 1 and 8 after sowing.

### Substrate Composition and Treatment

The growing medium was based on a mixture of white and black peat [80% and 20% (v/v), respectively, Klasmann-Deilmann, Geeste, Germany] with a bulk density of 298 g L^-1^ and an initial N concentration of 9 mg NH_4_^+^-N (L substrate)^-1^. It was limed according to the desired substrate pH levels. Besides the lime, the micronutrient fertilizer Radigen^®^ (Terraflor GmbH, Iserlohn, Germany) and phosphorus (P) fertilizers were mixed into the substrate twelve days before sowing. The final substrate mix was loosely stored in a bucket at 18°C–22°C with a moisture content adjusted to 49% and 47% (v/v) of the water capacity according to DIN EN 13041 (2012) in experiments 2 and 3, respectively. In experiment 1, substrates were used directly after mixing.

#### Adjustment of Initial Substrate pH

The substrate was limed with calcium carbonate (CaCO_3_) to set a substrate pH of 5.5 (**Table 1**) at the time of sowing. A substrate pH of 6.5 was realized by the addition of both CaCO_3_ and calcium oxide (CaO). By this means the conditions for a low as well as a high potential NH_3_ volatilization were realized.

#### Compost Amendment

In the third experiment, 5% (v/v) mature green waste compost was added to the peat substrate 1 day (treatment 9) or 12 days (treatment 8) before sowing (**Table 1**). The compost was characterized by a pH of 7.9 (CaCl_2_ extraction), a total water-soluble salt concentration of 1.28 g KCl (L substrate)^-1^ and a bulk density of 440 g (L substrate)^-1^. Nitrate and NH_4_^+^ concentrations in the compost were 22 and 97 mg N (L substrate)^-1^, respectively.

### Nutrient Demand and Supply

According to the recommendations of Lindner and Billmann (2006) a total requirement of 1,125 N, 196 P, and 448 potassium (K) mg per L substrate was estimated for the whole growing period of organically cultivated basil. The contents of plant-available nutrients in peat and compost were taken into account when calculating the fertilizer demand.

#### Nitrogen Supply

In the experiments 1 and 2, two organic N fertilization rates [250 and 750 mg N (L substrate)^-1^] in combination with two initial pH levels (5.5 and 6.5) were set up to realize different NH_3_ and NH_4_^+^ exposure levels (**Table 1**). The higher N dose corresponds to the upper fertilization range recommended for basil, which is fed solely by a base dressing. This limitation helps to prevent harmful effects on the seedling development due to high salt concentrations in the substrate, especially in the winter time (Eghbal, 2017). The lower N dose is common in organic basil production when the base dressing is complemented by repeated top dressings during the cultivation period.

Plant available N was supplied by a liquid amino acid fertilizer (AAF), manufactured from enzymatically hydrolyzed animal proteins [Fontana 9-0-0, MeMon B.V., Arnhem, Netherlands]. The batch used consisted of 8.8% (w/v) N and 54% (w/v) organic matter (OM). The C/N ratio in the fertilizer was 4. Previous incubation tests have shown that about 45% of the organic N was transformed to NH_4_^+^ within 5 days after mixing AAF into the peat substrate. Within an incubation time of 20 days no formation of NO_3_^-^ by nitrification was observed. Hence, AAF proved to be a suitable N source for providing high exposure with ammonical N forms (NH_4_^+^ and NH_3_) within the first weeks of plant cultivation. For base dressing, a nutrient solution containing AAF was supplied at sowing. In experiment 3, additional top dressings were necessary and realized by repeated substrate drenches with the AAF solution [125 mg N (L substrate)^-1^] at 45, 50, and 55 days after sowing. Corresponding to the plant fresh matter growth, which mainly determines the crop N requirement, between 250–375 mg N (L substrate)^-1^ were supplied by top dressings. No top dressing was applied when herb growth was severely restrained (**Table 1**). In a control treatment, which was included in all experiments, nitrogen was supplied by calcium nitrate tetrahydrate [Ca(NO_3_)_2_ • 4 H_2_O *pro analysi* (Merck KGaA, Darmstadt, Germany)] to achieve the maximum growth rate of basil under the respective trial conditions. From the literature, it is well known that the biomass production of basil is optimal with predominant NO_3_^-^-N supply (Kifferle et al., 2013; Frerichs et al., 2019). The total N fertilization rate in the control treatment was lower than in the organic treatments due to the fact that mineral N is completely phytoavailable whereas organic N fertilizers usually release not more than 60%–70% of their total N content as mineral N (NH_4_^+^ and NO_3_^-^) within a period of about 8 weeks (Hartz and Johnstone, 2006; Stadler et al., 2006).

#### Phosphorus (P) and Potassium (K) Supply

For P fertilization rock phosphate [12.7% (w/w) P], sieved with an ultra-centrifugal mill through a mesh screen of 200 μm, was used in the first two experiments. In the second experiment relatively small and dark green leaves were observed on plants of treatment 4 with an initial substrate pH of 6.5 and a base dressing of 250 mg N (L substrate)^-1^. These symptoms might indicate the occurrence of P deficiency (Gibson et al., 2007). Therefore, in the third experiment the type of P fertilizer was changed to a micro-granulated bone meal [DCM ECO-FOS, 10.0% (w/w) P, 4.0% (w/w) N, Deutsche CUXIN Marketing GmbH, Otterndorf, Germany]. This P fertilizer is often used in organic basil production and was considered to maintain a sufficient P availability under these cultivation conditions. Potassium was supplied as sulfate (K_2_SO_4_ *pro analysi*, Merck KGaA, Darmstadt, Germany) *via* substrate drenches on the day of sowing.

#### Additional Nutrient Supply

To alleviate P deficiency, which appeared in the control treatment of experiment 3, all plants in this experiment received an additional drench with calcium dihydrogen phosphate monohydrate [Ca(H_2_PO_4_)_2_ • H_2_O] at a dosage of 17.4 mg P (L substrate)^-1^ 33 days after sowing. In experiment 1, young leaves of basil plants in treatment 5 [initial substrate pH 6.5; 750 mg AAF-N (L substrate)^-1^] showed yellowish discoloration in the last days of cultivation. These symptoms may indicate the occurrence of iron (Fe) deficiency (Bergmann, 1993; Gibson et al., 2007). To avoid related growth limitations, micronutrient supply was complemented 2 and 3 weeks after sowing by overhead irrigation, each time with 50 ml per pot of 0.25 g L^-1^ Flory 10 (ProfiFlor GmbH, Pulheim, Germany) in experiments 2 and 3, respectively.

### Substrate Analyses and Measurement of Plant Parameters

For the preparation of substrate samples, the entire substrate of two pots per plot and sampling date were homogenized and an aliquot portion of it was used for analysis. Substrate samples from organically fertilized treatments were taken on a weekly basis to determine NH_4_^+^, NO_3_^-^, and NO_2_^-^ concentrations (extracted with 0.0125 M CaCl_2_) as well as the substrate pH (0.01 M CaCl_2_). The control treatments were sampled at the beginning and end of the trial period, in experiment 3 also at an intermediate date. Total water-soluble salt concentration was measured in experiment 1. In experiment 3, calcium chloride/DTPA (CAT) extractable P concentration was quantified. All substrate sample preparations were performed in accordance with guidelines of the Association of German Agricultural Analytic and Research Institutes e. V. (VDLUFA) (Hoffmann, 1997). The subsequent substrate analyses were conducted by using ion-selective electrodes to detect NH_4_^+^, substrate pH, and total water-soluble salt concentration (Thermo Orion Standard Ammonia Electrode, Thermo Electron corporation, USA; Feld-pH-Meter, pH/cond 340i, Xylem Analytic Germany Sales GmbH & Co. KG, WTW, Weilheim, Germany). For NH_4_^+^ detection, soil extract was buffered to pH > 11 by 2% ammonia ionic strength adjuster (Thermo Fisher Scientific, USA). Ion chromatography was used to detect NO_3_^-^ (Compact IC plus 882, Deutsche Metrohm GmbH Co. KG, Filderstadt, Germany), reflectometry to detect NO_2_^-^ (RQflex^®^ plus 10 and Reflectoquant^®^ test strips, Merck KGaA, Darmstadt, Germany), as well as spectrophotometry (Specord 40-400189, Analytik Jena AG, Jena, Germany) to detect PO_4_^3-^ based on the molybdenum blue reaction according to VDLUFA method A 13.1.1 (Hoffmann, 1997). Plant development was monitored by determining the number of plants, shoot fresh matter yield, and plant height (distance from substrate surface to the tip of the longest shoot) in each pot.

### Aerial NH_3_ Measurements and Related Methodological Evaluations

**Figure 1:**
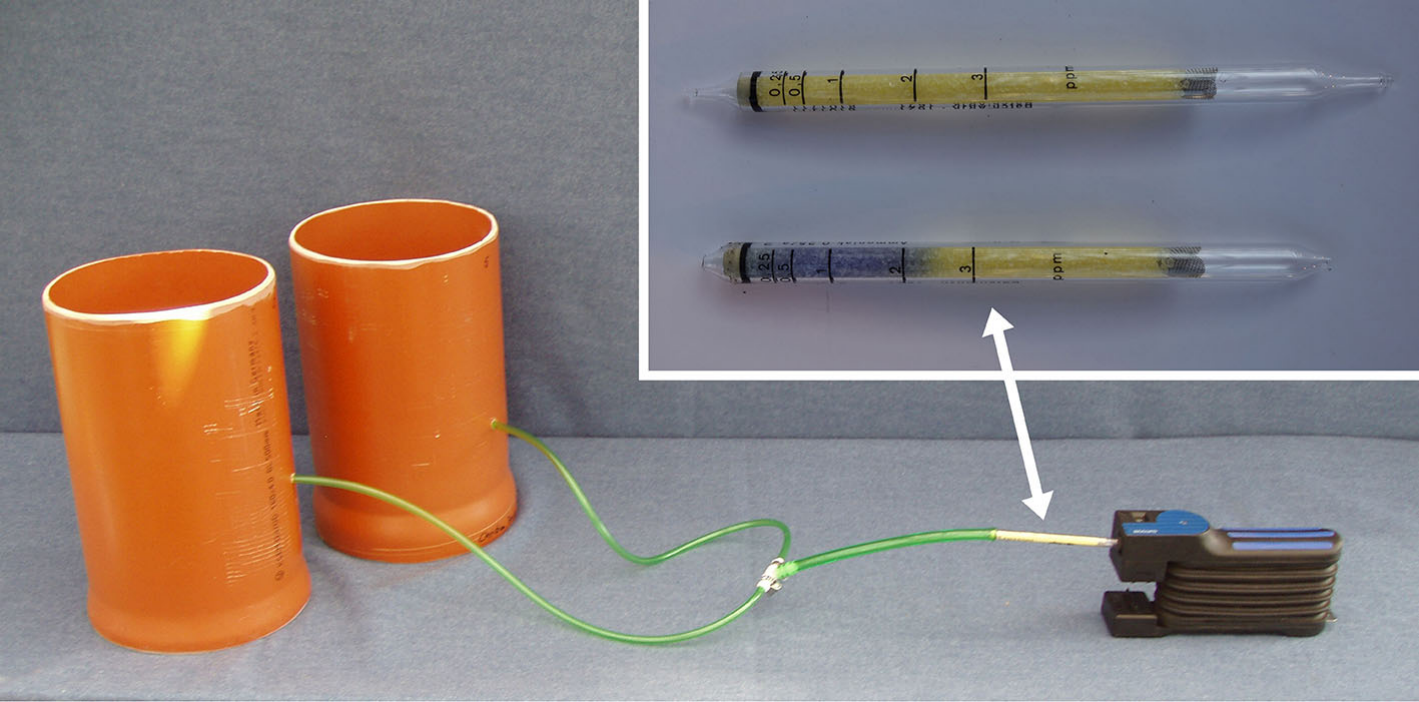
Ammonia measurement system consisted of open-top chambers which were connected with NH_3_ sampling tubes, NH_3_ detector tube, and manual bellows pump.

**Figure 2:**
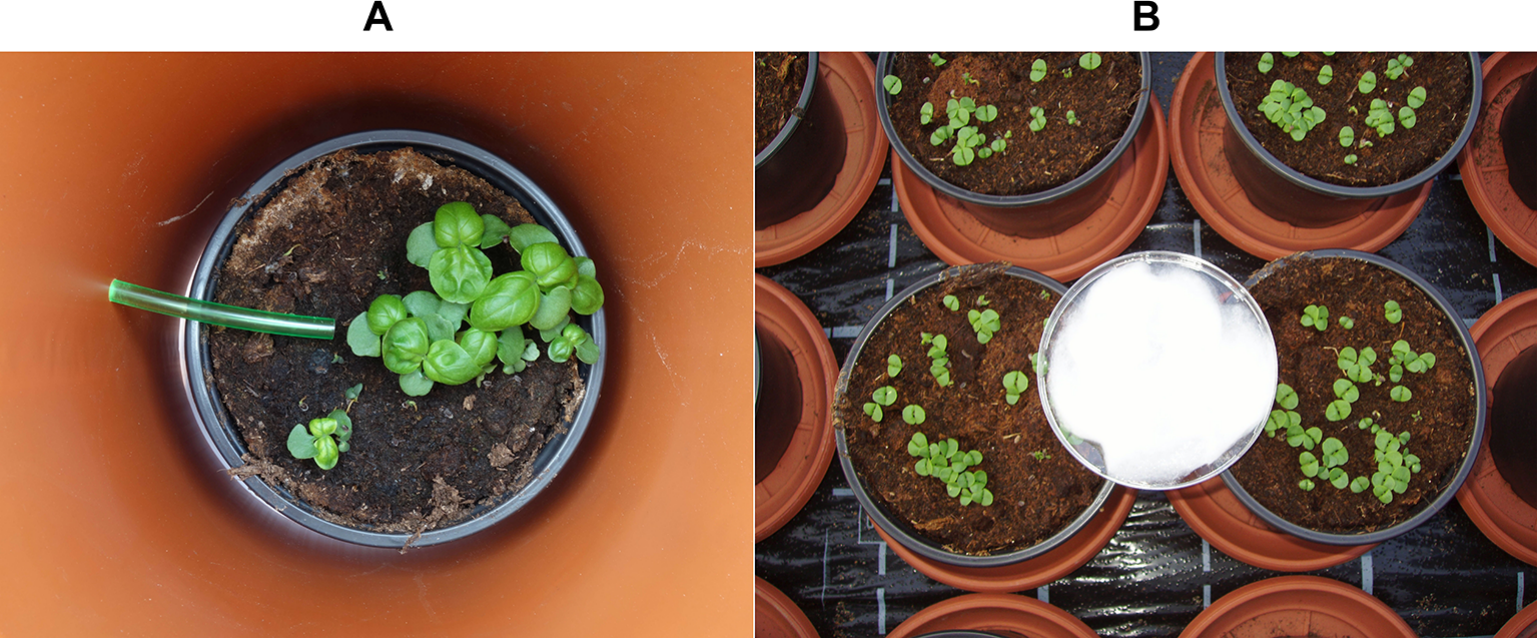
Ammonia sampling tube placed on the substrate surface inside the open-top chamber **(A)**; Acid trap used as passive NH_3_ samplers (plastic bowls with viscose pad) **(B)**.

The NH_3_ concentration in the aerial environment of pot-grown basil was determined by using an open-top chamber approach. The basic design and operation principles were adapted from a field measurement system described by Pacholski et al. (2006). Each measuring unit consisted of two chambers made from polyethylene (PE) pipes. Both chambers were connected by flexible PE tubes fixed by a manifold to a manual bellows pump (Accuro-Balgpumpe, Drägerwerk AG & Co. KGaA, Lübeck, Germany), which served as an air sample device (**Figure 1**). For NH_3_ measurement, one basil pot was transferred in each chamber. The inlet side of the tubes was placed on the substrate surface (**Figure 2A**). Air samples were taken by using the hand pump and passed through a NH_3_ detector tube (Dräger-Tube^®^ Ammoniak 0.25/a, Drägerwerk AG & Co. KGaA, Lübeck, Germany) (**Figure 1**). By 10 pumping strokes, which require about 1 min, 1,000 ml air was sucked through the pump and the indicator tube. The NH_3_ concentration in the sampled air was immediately displayed on the scale of the detector tube by a blue reaction product. The readings were corrected afterwards to standard atmospheric pressure (1,013 hPa). Ammonia measurements took place twice a day: at sunrise and in the early afternoon to detect NH_3_ at the lowest and highest daily temperature, respectively. Both measurements were averaged for further data analysis. With the described procedure, NH_3_ concentrations between 0.250–3.000 ppm were detectable. By doubling the number of pumping strokes to 20, it was possible to achieve a measuring range of 0.125–1.500 ppm. To avoid cross contamination when performing NH_3_ measurements, separate chamber systems for each treatment were used. Likewise, before each measurement, the sampling tubes were flushed with 500 ml air.

Preliminary investigations focusing on the reproducibility of the NH_3_ measurements have shown that the coefficient of variation ranged from 8% to 12% when NH_3_ concentration exceeded 0.250 ppm. At lower NH_3_ concentrations, the coefficient of variation increased substantially. Under constant conditions in terms of temperature and irradiation the placing time (0–15 min) of the pots in the open-top chamber did not affect the detected NH_3_ concentration. For further methodological evaluations, NH_3_ enrichment in the canopy environment of basil was determined by two additional methods: a) acid traps used as passive NH_3_ samplers and b) calculation approach based on the NH_3_/NH_4_^+^ equilibrium in the solution of the growing medium. The acid traps were made similar as described by Shigaki and Dell (2015) from plastic bowls (diameter: 10 cm) which were filled with 0.05 M H_2_SO_4_. To absorb atmospheric NH_3_, the traps were positioned for 24 h on the substrate surface (**Figure 2B**). A viscose pad placed in the plastic bowl was used to enhance the absorption capacity by a thicker surface layer. In the acid solution, NH_3_ was converted immediately to NH_4_^+^. After dilution with demineralized water, NH_4_^+^ was detected by reflectometry (RQflex^®^ plus 10 and Reflectoquant^®^ test strips, Merck KGaA, Darmstadt, Germany). The calculation approach took into account the parameters air temperature, NH_4_^+^ concentration, and pH of the peat substrate, which were determined in parallel with NH_3_ measurements. The theoretically formed NH_3_ concentration in the solution of growing medium was estimated according to Hobiger (1996). Results obtained from both comparative methods were linearly correlated to the NH_3_ concentrations determined with the open-top chamber technique as indicated by coefficients of determination (*R* *²*, *P* < 0.001) of 0.75 (acid traps) and 0.67 (calculation approach).

NH_3_ concentration in air samples of the control treatments (NO_3_^-^ fertilization) was always below detection limit, even when NH_3_ concentration in neighboring plots reached a maximum level of 1.8 ppm. In contrast, in acid traps placed on NO_3_^-^-fed basil pots (control) a noticeable NH_4_^+^ accumulation was measured, which reached up to one third of the level in the organically fertilized plots. Therefore, we assume that the open-top chamber method used in our experiments was appropriate to prevent overlapping effects by NH_3_ diffusion and drift processes within the arranged multi-plot set-up.

### Statistical Analysis

Plant growth parameters were analyzed by ANOVA and Tukey’s *post hoc* test (*P* < 0.05). Beforehand, assumptions of normality and homogeneity of variances were tested according to the Kolmogorov-Smirnov test and the Fmax test (Köhler et al., 2012), respectively. If needed, data were logarithmically transformed to meet normal distribution and homogeneity of variance. In experiments 1 and 2, the organic treatments were analyzed by two-way ANOVA (initial substrate pH x N base dressing rate). To compare the organic treatments with the NO_3_^-^-fed control, a one-way model was used. Likewise, experiment 3 was analyzed by one-way ANOVA. Relationships between substrate properties and plant growth performance were examined by linear regression analysis. The same approach was used to assess the effects of the aerial NH_3_ concentration on basil. All analyses were performed using the statistical software SPSS, version 25 (IBM Deutschland GmbH, Ehningen, Germany).

## Results

### Development of Substrate Parameters and Aerial NH_3_ Concentration

#### Substrate NH_4_^+^ Concentration

**Figure 3:**
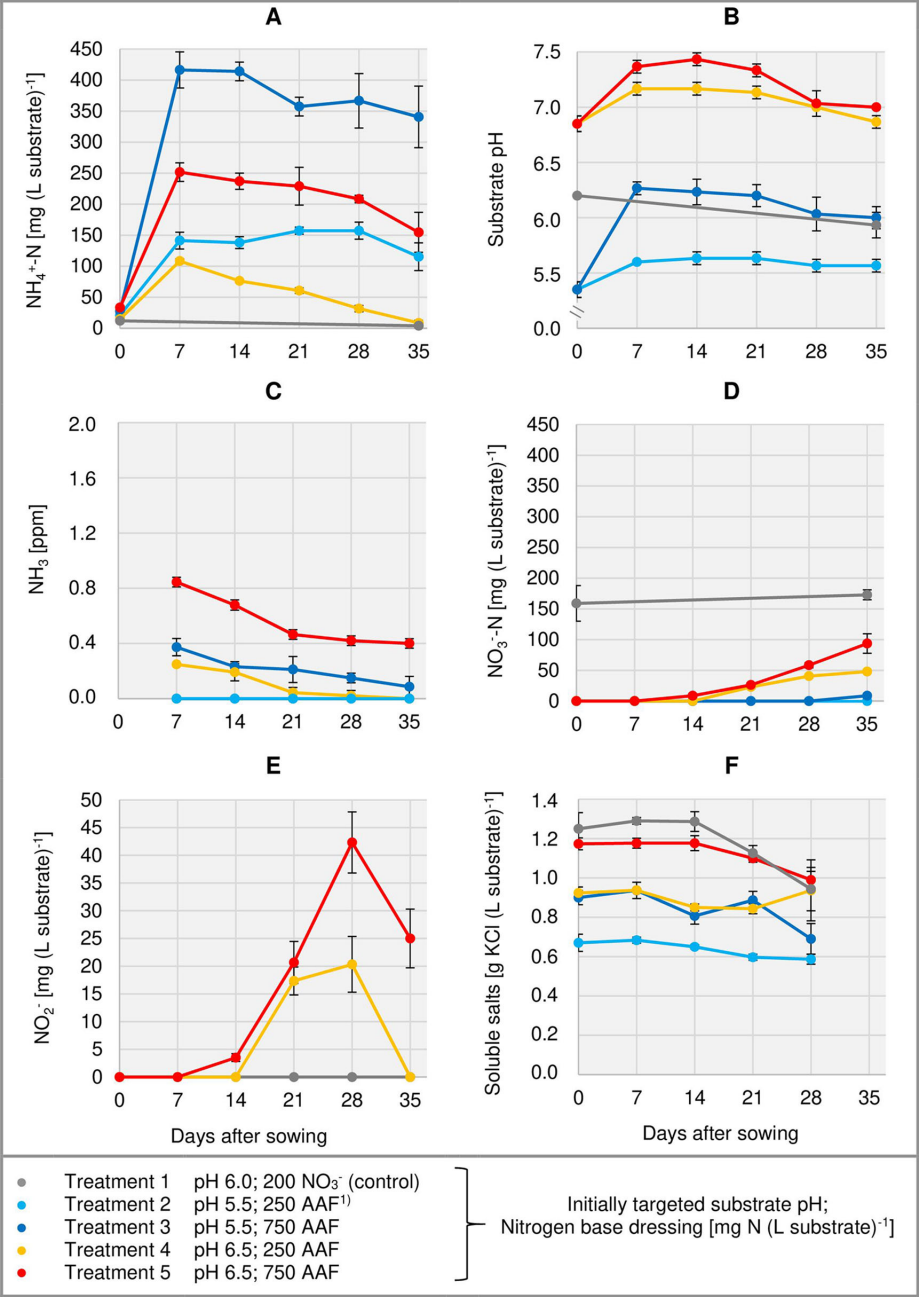
Development of substrate NH_4_^+^-N **(A)**, NO_3_^-^-N **(D)**, and NO_2_^-^ **(E)** concentration as well as substrate pH **(B)** and NH_3_ concentration in the aerial environment of basil plants **(C)** over time in experiment 2. Total water-soluble salt concentration of the substrate in experiment 1 is shown in **(F)**. Error bars indicate standard deviation (*n* = 3). ^1)^AAF, amino acid fertilizer (main component of the organic N supply).

**Figure 4:**
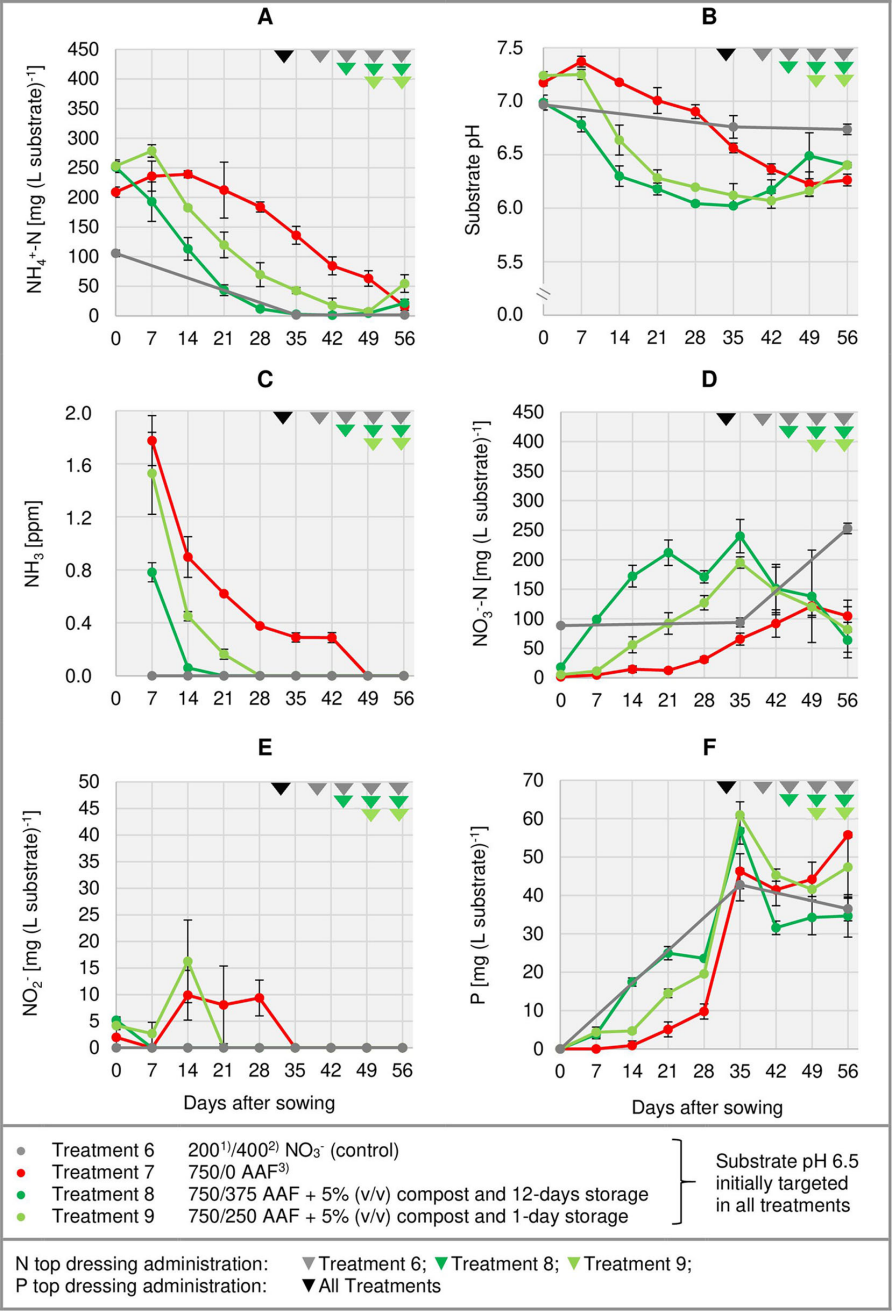
Development of substrate NH_4_^+^-N **(A)**, NO_3_^-^-N **(D)**, NO_2_^-^ **(E)**, and P **(F)** concentration as well as substrate pH **(B)** and NH_3_ concentration in the aerial environment of basil plants **(C)** over time in experiment 3. Error bars indicate standard deviation (*n* = 3). ^1)^N base dressing/ ^2)^N top dressing [mg N (L substrate)^-1^]; ^3)^AAF, amino acid fertilizer (main component of the organic N supply).

Right from the beginning of basil cultivation, NH_4_^+^ concentration rose in all treatments fertilized with an amino acid fertilizer (AAF). In experiment 2, 7 days after sowing approximately 56% of the supplied organic N was detected in the form of NH_4_^+^ when substrate pH was initially adjusted to 5.5. In contrast, at pH 6.5 only 34%–43% of the supplied organic N appeared as NH_4_^+^ (**Figure 3A**). In experiment 3, already on the day of sowing a high concentration of NH_4_^+^ was available in the growing medium (**Figure 4A**). Without compost amendment, the NH_4_^+^ concentration remained at the same level for about 4 weeks and then decreased continuously until the end of the experiment. In the peat-compost mixtures the nitrification process was accelerated and thus NH_4_^+^-N concentration decreased below 100 mg (L substrate)^-1^ already 3–4 weeks after sowing. In experiment 3, the top dressings slightly increased the substrate NH_4_^+^ concentration in the last 2 weeks of cultivation.

#### Substrate pH

In the first week after sowing, substrate pH increased by 0.5–1.0 unit in organically fertilized treatments without compost amendment (**Figures 3B**, **4B**). Afterwards, substrate pH remained on a slightly alkaline level for about 4 weeks when initially set to pH 6.5. Subsequently substrate pH decreased steadily. In experiment 3, which had the longest experimental period (8 weeks of cultivation), pH reached 6.3. In pots amended with compost, substrate pH dropped below 7.0 already 1 or 2 weeks after sowing when using a pre-storage treatment of 12 days or 1 day, respectively. After a top dressing of AAF, substrate pH slightly increased from 6.0 to maximum 6.5 in the last 2 weeks of cultivation.

#### NH_3_ Exposure in the Aerial Environment of Basil

Under high NH_4_^+^ concentrations and slightly alkaline conditions in the substrate, NH_3_ concentration in the canopy airspace of basil ranged initially between 0.4 and 1.8 ppm (**Figures 3C**, **4C**). In the following weeks, NH_3_ concentration declined continuously even when NH_4_^+^ was still the predominant N source in the growing medium and substrate pH was above 7.0 (treatments 4, 5, and 7). In the first two experiments, NH_3_ remained below the detection limit (< 0.125 ppm) throughout the whole crop period when the base dressing rate was limited to 250 mg AAF-N (L substrate)^-1^ and substrate pH was initially adjusted to 5.5. Likewise, with a straight NO_3_^-^ supply (treatments 1 and 6) no NH_3_ was detected. The addition of 5% (v/v) mature compost to the organically fertilized peat accelerated the decrease of the NH_3_ concentration, especially when the mixed growing medium was previously stored for 12 days (treatment 8). In this case, NH_3_ had almost completely disappeared 2 weeks after sowing. However, when the compost-amended peat was stored for just 1 day (treatment 9) this NH_3_ depletion was delayed by 1 week. Repeated top dressings of AAF-N (treatments 8 and 9) applied during the last 11 days of cultivation did not stimulate NH_3_ volatilization from the substrate. Aerial NH_3_ concentration was always below the detection limit in this period.

#### Substrate NO_3_^-^ Concentration and NH_4_^+^-N/NO_3_^-^-N Ratio

The occurrence of NO_3_^-^ in organically fertilized peat was also affected by the initial substrate pH. At pH 5.5, the NO_3_^-^ concentration remained below 10 mg N (L substrate)^-1^ for the entire trial period (**Figure 3D**). In contrast, with an initial pH of 6.5 the NO_3_^-^ concentration rose above 50 mg N (L substrate)^-1^. Nevertheless, even here 4–5 weeks had elapsed before this level was reached. Therefore, at the seedlings stage (7–21 days after sowing), basil was exposed to a high NH_4_^+^-N/NO_3_^-^-N ratio. A much faster NO_3_^-^ accumulation was observed when peat was mixed with mature compost and subsequently stored for 12 days before use (**Figure 4D**; **Table 4**). In this substrate, the NH_4_^+^-N/NO_3_^-^-N ratio was nearly balanced at the basil seedlings stage about 10 days after sowing. Another week later NO_3_^-^ was the predominant N source in the substrate. If the storage time of the peat-compost blend was limited to 1 day, NO_3_^-^ accumulation lagged 2 weeks behind and was less pronounced. At the end of the cultivation period, NO_3_^-^ concentration in organically fertilized treatments ranged between 50 and 100 mg N (L substrate)^-1^. A considerably higher level of NO_3_^-^ was detected in the substrate of NO_3_^-^-fed basil.

#### Substrate NO_2_^-^ Concentration

In addition to NH_4_^+^ and NH_3_, NO_2_^-^ is also a potentially phytotoxic N species. Its concentration in the organically fertilized substrate ranged between 0 and 42 mg (L substrate)^-1^ and peaked 2–4 weeks after sowing (**Figures 3E**, **4E**) with the onset of an enhanced nitrification (**Figures 3D**, **4D**). In compost-amended peat NO_2_^-^ accumulation was suppressed when the prepared growing medium was stored for 12 days. Likewise, NO_2_^-^ concentration remained always below 2.5 mg (L substrate)^-1^ (limit of quantification) in the control treatment with NO_3_^-^ supply.

#### Total Water-Soluble Salt and CAT-Extractable P Concentrations of the Substrate

Total water-soluble salt concentration of the substrate (expressed as KCl) was measured in experiment 1 and ranged from 0.6 to 1.3 g (L substrate)^-1^ (**Figure 3F**). In experiment 3, concentration of CAT-extractable P amounted to ≤ 25 mg (L substrate)^-1^ during the first 4 weeks of cultivation. After a top-dressing with calcium dihydrogen phosphate CAT-extractable P concentration rose and remained above 30 mg P (L substrate)^-1^ until the end of the cultivation period (**Figure 4F**).

### Effect of NH_3_ and NH_4_^+^ Exposure on the Growth of Basil

#### Germination of Basil

**Figure 5:**
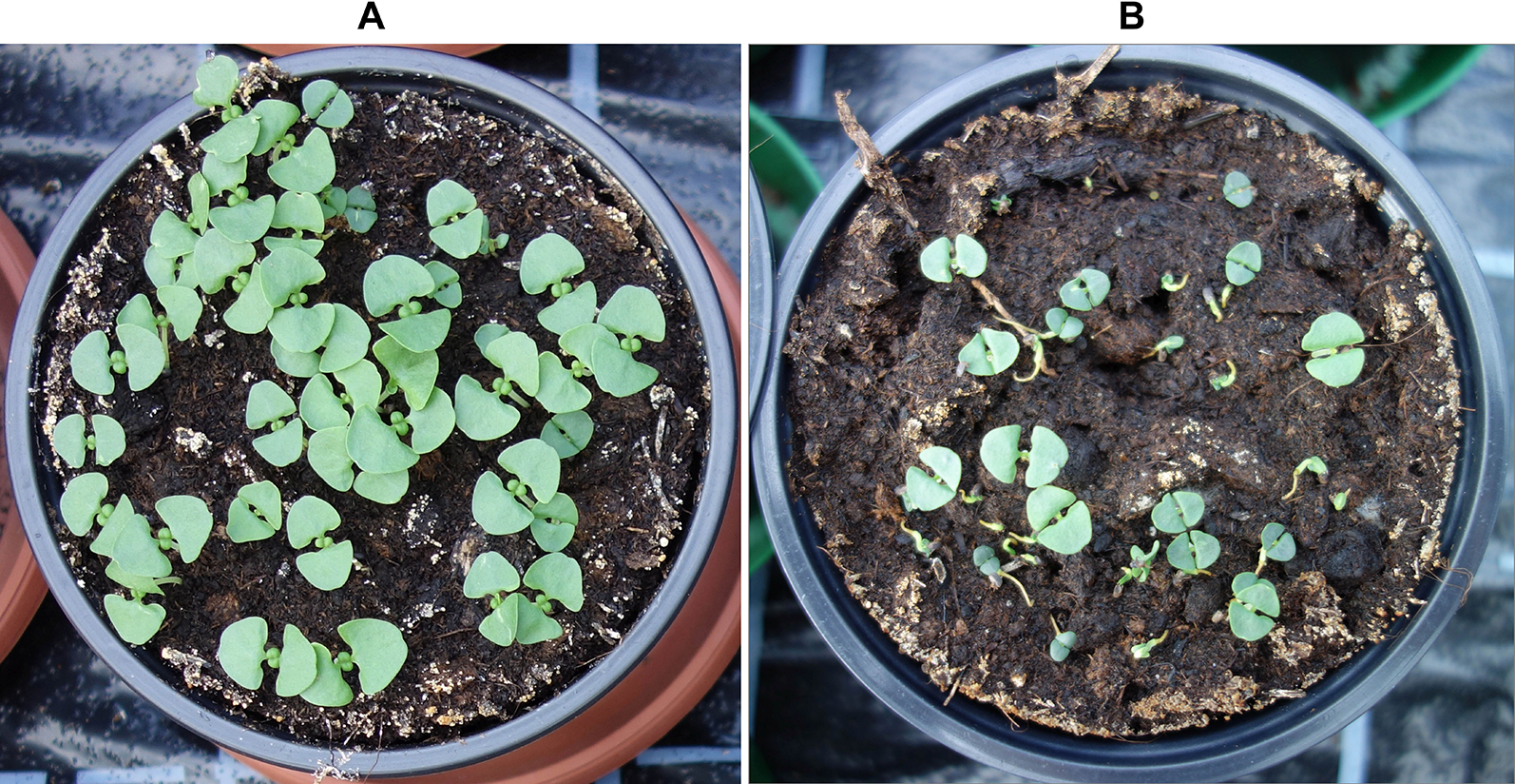
Typical basil seedlings 14 days after sowing in the NO_3_^-^-fed control **(A)** and exposed to high NH_3_ and NH_4_^+^ concentrations in treatment 7 **(B)**.

**Figure 6:**
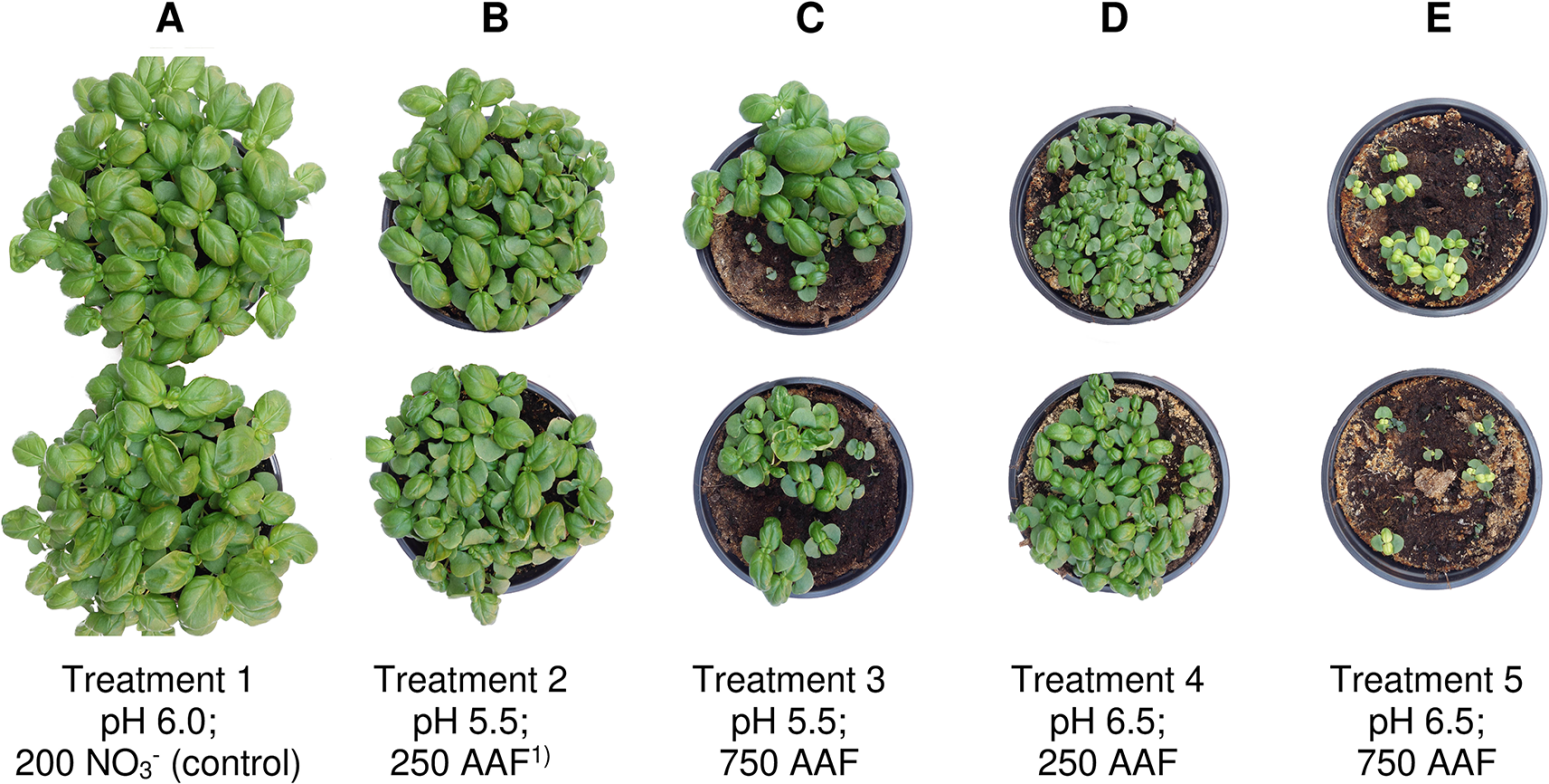
Basil plants 35 days after sowing in experiment 2. Nitrate-fed control **(A)**; base dressing 250 mg AFF-N (L substrate)^-1^ **(B, D)** and 750 mg AFF-N (L substrate)^-1^ **(C, E)**; initial substrate pH 5.5 **(B, C)** and 6.5 **(D, E)**. ^1)^AAF, amino acid fertilizer (main component of the organic N supply).

Within the first week after sowing, radicles and germ buds emerged from seeds. In all treatments, a homogeneous onset of germination was observed. However, subsequently seedling growth was stunted in treatments exposed to high NH_3_ and NH_4_^+^ concentrations (**Figure 5**). The elongation of the radicle was inhibited and thus it did not grow into the substrate. Likewise, the development of hypocotyl and cotyledons was impaired. In addition, the primary leaves became partially chlorotic (**Figure 6E**). However, cotyledons stayed green and were free from necrotic symptoms. Three weeks after sowing, most of the stunted seedlings were dead. From the fourth week of cultivation, the number of plants remained constant. Therefore, results presented in **Tables 2**–**6** consider the mean NH_3_ and NH_4_^+^ concentration in the juvenile stage of basil (7–21 days after sowing).

**Table 2:** Number of plants, fresh matter yield and plant height 28 and 35 days after sowing as affected by exposure to ammonical N forms determined within 7–21 days after sowing in experiments 1 and 2, respectively (*n* = 3).

Treatment^1)^	Mean exposure (7–21 days after sowing)		Yield parameter			
	NH_3_[ppm]	NH_4_^+^-N [mg L (substrate)^-1^]	Number of plants [% of control]	Fresh matter yield [g pot^-1^]	Plant height [cm]
**Experiment 1**					
1 (control) ^2)^ (pH 6.0/200 NO_3_^-^-N)	< 0.1 c	10 e	100 a	10.8 a	5.5 a
2 (pH 5.5/250 AAF-N)	< 0.1 c	83 c	96 ab	7.2 b	3.8 b
3 (pH 5.5/750 AAF-N)	0.2 b	279 a	91 ab	6.1 bc	3.6 b
4 (pH 6.5/250 AAF-N)	0.1 bc	55 d	96 ab	3.1 cd	2.5 c
5 (pH 6.5/750 AAF-N)	0.6 a	148 b	88 b	2.5 d	2.5 c
**Experiment 2**					
1 (control) ^3)^ (pH 6.0/200 NO_3_^-^-N)	< 0.1 c	< 5 e	100 a	10.6 a	6.1 a
2 (pH 5.5/250 AAF-N)	< 0.1 c	146 c	91 a	8.9 b	5.6 a
3 (pH 5.5/750 AAF-N)	0.3 b	396 a	66 b	3.6 c	4.2 b
4 (pH 6.5/250 AAF-N)	0.2 bc	82 d	88 a	3.9 c	3.6 b
5 (pH 6.5/750 AAF-N)	0.7 a	239 b	30 c	0.7 d	2.1 c

^1)^Initially targeted substrate pH/Base dressing [mg N (L substrate)^-1^].

^2)^Mean concentrations detected 0 and 28 days after sowing.

^3)^Mean concentrations detected 0 and 35 days after sowing.

Means within a column not sharing a letter are significantly different according to Tukey’s test (*P* < 0.05).

**Table 3:** Analysis of variance results for effects of initial substrate pH and N base dressing on number of plants, fresh matter yield, and plant height in experiments 1 and 2 (28 and 35 days after sowing, respectively).

Source of variance	Experiment 1			Experiment 2		
	Number of plants	Fresh matter yield	Plant height	Number of plants	Fresh matter yield	Plant height
	[% of control]	[g pot^-1^]	[cm]	[% of control]	[g pot^-1^]	[cm]
Initial substrate pH	n.s.	**	**	**	***	***
N base dressing	*	n.s.	n.s.	***	***	***
Interaction	n.s.	n.s.	n.s.	**	**	**
***R^2^*** ** for a linear regression model**						
NH_4_^+^ concentration	(-) 0.23 ^n.s.^	(-) 0.01 ^n.s.^	(-) 0.04 ^n.s.^	(-) 0.23 ^n.s.^	(-) 0.10 ^n.s.^	(-) 0.01 ^n.s.^
Substrate pH	(-) 0.07 ^n.s.^	(-) 0.80 ***	(-) 0.86 ***	(-) 0.33 ^n.s.^	(-) 0.70 **	(-) 0.81 ***
NH_3_ concentration	(-) 0.36 *	(-) 0.35 *	(-) 0.30 ***	(-) 0.93 ***	(-) 0.92 ***	(-) 0.80 ***

Significance level: n.s., not significant; *P < 0.05; **P < 0.01; ***P < 0.001.

Lower section of the table: Relationships between plant growth parameters and mean exposure to ammonical N forms as well as substrate pH in the organically fertilized treatments determined within 7–21 days after sowing indicated by direction (+/-) and coefficient of determination (R*^2^*) for a linear regression model (n = 12).

**Table 4:** NH_3_, NH_4_^+^-N, and NO_3_^-^-N concentration as well as NH_4_^+^-N/NO_3_^-^-N ratio determined within 7–21 days after sowing in experiment 3 (*n* = 3).

Treatment^1)^	Mean exposure (7–21 days after sowing)			
	NH_3_ [ppm]	NH_4_^+^-N [mg L (substrate)^-1^]	NO_3_^-^-N [mg L (substrate)^-1^]	NH_4_^+^-N/NO_3_^-^-N ratio
6 (control) ^2)^ (NO_3_^-^-N, 200/400)	< 0.1 d	54 d	91 b	0.6 c
7 (AAF-N, 750/0)	1.1 a	229 a	10 d	68.8 a
8 (AAF-N, 750/375, 5% compost, 12-day storage)	0.3 c	116 c	161 a	0.9 c
9 (AAF-N, 750/250, 5% compost, 1-day storage)	0.7 b	194 b	53 c	9.9 b

^1)^Fertilized N form, base dressing/top dressing rate [mg N (L substrate)^-1^], compost addition, and storage period of the prepared substrate before use; substrate pH was initially adjusted to 6.5 in all treatments of this experiment.

^2)^Mean concentrations detected 0 and 35 days after sowing.

Means within a column not sharing a letter are significantly different according to Tukey’s test (P < 0.05).

**Table 5:** Number of plants, fresh matter yield, and plant height 35 days and 56 days after sowing in experiment 3 (*n* = 3).

Treatment^1)^	35 days after sowing			56 days after sowing	
	Number of plants	Fresh matter yield	Plant height	Fresh matter yield	Plant height
	[% of control]	[g pot^-1^]	[cm]	[g pot^-1^]	[cm]
6 (control) (NO_3_^-^-N, 200/400)	100 a	3.4 b	2.2 bc	27.5 b	9.7 c
7 (AAF-N, 750/0)	39 c	0.7 c	1.1 c	13.0 c	7.5 c
8 (AAF-N, 750/375, 5% compost, 12-day storage)	68 b	9.2 a	6.1 a	54.3 a	23.3 a
9 (AAF-N, 750/250, 5% compost, 1-day storage)	35 c	2.0 b	2.8 b	31.8 b	15.3 b
***R^2^*** ** for a linear regression model^2)^**					
NH_4_^+^ concentration	(-) 0.69 **	(-) 0.91 ***	(-) 0.92 ***	(-) 0.95 ***	(-) 0.88 ***
Substrate pH	(-) 0.42 ^n.s.^	(-) 0.90 ***	(-) 0.85 ***	(-) 0.95 ***	(-) 0.91 ***
NH_3_ concentration	(-) 0.55 *	(-) 0.97 ***	(-) 0.94 ***	(-) 0.98 ***	(-) 0.95 ***
NO_3_^-^ concentration	(+) 0.63 *	(+) 0.94 ***	(+) 0.94 ***	(+) 0.93 ***	(+) 0.86 ***
NH_4_^+^–N/NO_3_^-^-N ratio	(-) 0.25 ^n.s.^	(-) 0.47 *	(-) 0.49 *	(-) 0.64 *	(-) 0.61 ***

^1)^Fertilized N form, base dressing/top dressing rate [mg N (L substrate)^-1^], compost addition, and storage period of the prepared substrate before use; substrate pH was initially adjusted to 6.5 in all treatments of this experiment.

^2)^Significance level: n.s., not significant; *P < 0.05; **P < 0.01; ***P < 0.001.

Means within a column not sharing a letter are significantly different according to Tukey’s test (P < 0.05). Lower section of the table: Relationships between plant growth parameters and exposure to mineral N forms as well as substrate pH in the organically fertilized treatments, determined within 7–21 days after sowing, indicated by direction (+/-) and coefficient of determination (R*^2^*) for a linear regression model (n = 9).

**Table 6:** Maximum ammonical N exposure determined on average 7–21 days after sowing to generate ≥ 90% of plant yield performance, compared to the NO_3_^-^-fed control (28 and 35 days after sowing in experiments 1 and 2, respectively) (*n* = 30).

Parameter	Ammonical exposure 7–21 days after sowing		
	NH_3_ exposure [ppm]	NH_4_^+^-N Concentration [mg L (substrate)^-1^]^*^	
		With NH_3_ exposure ≥ 0.2 ppm	With NH_3_ exposure < 0.2 ppm
Number of plants	< 0.2	< 100	< 300
Fresh matter yield	< 0.1	< 50	< 50
Plant height	< 0.1	< 50	< 50

*Substrate contained less than 15 mg NO_3_^-^-N L^–1^.

Compared to the NO_3_^-^-fed control, the number of plants was reduced by up to 70% in treatments with a base dressing of 750 mg AFF-N (L substrate)^-1^ at an initial substrate pH of 6.5 (**Tables 2**, **5**). The emergence of healthy plants was improved when substrate pH was initially adjusted to 5.5 and, to a much greater extent, by decreasing the base dressing rate to 250 mg AFF-N (L substrate)^-1^. In this case, plant emergence rate was not negatively affected even at a higher pH level. Likewise, the addition of mature compost to peat substantially enhanced the proportion of healthy plants if the amended substrate was subjected to previous storage for 12 days. The number of plants was positively correlated to the NO_3_^-^ concentration and negatively correlated to the NH_4_^+^-N/NO_3_^-^-N ratio determined in the growing medium during the juvenile stage of plant growth (**Table 5**). When the ammonical N concentration exceeded 100 mg NH_4_^+^-N (L substrate)^-1^ and 0.2 ppm NH_3_ in the aerial plant environment, the number of plants was reduced by more than 10% compared to the NO_3_^-^-fed control (**Table 6**). However, the adverse effect of NH_4_^+^ on seed germination was less pronounced as long as the NH_3_ concentration remained ≤ 0.2 ppm.

#### Plant Shoot Biomass Production and Plant Height

Growth impairment at the juvenile stage had a lasting negative impact on crop development. Shoot fresh matter yield was strongly reduced at the end of the experiments when basil grown in pure peat was fertilized with 750 mg AAF-N (L substrate)^-1^ and/or substrate pH was adjusted to 6.5 before use (**Tables 2** and **5** and **Figures 6C**–**E**). ANOVA showed that crop yield was not significantly related to the number of plants (*P* ≥ 0.25). Thus, biomass production of organically fertilized basil was adversely affected even beyond the impact of the reduction in plant numbers. Shoot elongation was also inhibited, as indicated by the decreased plant height. Furthermore, leaf blades remained smaller (**Figures 6D, E**).

Both plant height and fresh matter yield of organically fertilized basil were negatively correlated to NH_3_ and NH_4_^+^ exposure in the early cultivation period. Furthermore, plant growth was reduced by increasing substrate pH (**Tables 3** and **5**). Conversely, rising NO_3_^-^ supply and declining NH_4_^+^-N/NO_3_^-^-N ratio promoted crop performance (**Table 5**). Without noticeable NO_3_^-^ formation in the first 3 weeks of cultivation, maximum growth of organically fertilized basil was observed with a base dressing of 250 mg AAF-N (L substrate)^-1^ in combination with an initial substrate pH of 5.5 (**Figure 6B**). In this treatment NH_4_^+^ remained relatively constant at about 150 mg N (L substrate)^-1^ (**Figure 3A**). With this N supply fresh matter yield and plant height were reduced by 8%–34% compared to the NO_3_^-^-fed control in the first two experiments (**Table 2**). In all other organic N fertilization treatments fresh matter yield and plant height were reduced by 44%–94% and 31%–66% respectively.

In experiment 3, maximum plant growth was observed when basil was grown in a compost-amendment peat that had previously been stored for 12 days (**Table 5**). In this treatment basil reached a plant height of 15 cm, which is required for marketing of the produce, 7 weeks after sowing. However, when the prepared compost-peat substrate was stored for just 1 day, the marketable crop size was achieved with a delay of 1 week (data not shown). Without compost addition plant height reached to 7.5 cm after eight weeks of cultivation, compared to 9.7 cm for the control. The relatively weak growth performance of NO_3_^-^-fed plants in this experiment, was related to a temporary shortage in P supply during the first half of cultivation. Phosphorus deficiency symptoms became visible in the control treatment about 4 weeks after sowing, when cotyledons turned purple and shoot elongation lagged behind. Although a substrate drench with a water-soluble P fertilizer was conducted soon afterwards, plant growth remained distinctly lower than usually observed with N supplied as NO_3_^-^ (e.g., in experiment 2, **Figure 6A**).

## Discussion

### N Dynamic in the Substrate After Organic N Fertilization

Basil reaches its marketable size in greenhouse pot cultivation 4–10 weeks after sowing (Eghbal, 2017). To ensure sufficient N supply in organic production, the crop should be fertilized with easily decomposable organic N sources. The mineralization of the AAF investigated in this study started immediately after its incorporation into the growing medium, as indicated by the rapidly increasing concentration of NH_4_^+^ (**Figures 3A**, **4A**) and rising substrate pH (**Figures 3B**, **4B**) in the first week of cultivation. However, nitrification was delayed by 3–4 weeks in treatments with peat as sole substrate component. Comparable observations were made after application of different organic fertilizers such as horn shavings, blood meal, and urea to peat or wood-based substrates (Niemiera et al., 2014; Frerichs et al., 2017; Bergstrand et al., 2018). After adding the amino acid arginine to soil samples, Kemmitt et al. (2006) detected the highest NH_4_^+^ concentration 2–8 days later. The depletion of the amino acid pool was accelerated by liming of the natively acid soils. The correlation between the N mineralization rate and soil pH could be described by a quadratic equation that indicates a maximum NH_4_^+^ accumulation at pH 5.5. Similarly, in our experiments we always observed a considerably higher NH_4_^+^ concentration in peat substrates initially adjusted to pH 5.5 rather than to pH 6.5 (**Figure 3A**). In contrast to these findings it is well-known that most soil microorganisms prefer soil pH around 7.0 (Coyne, 1999). Thus, in several laboratory soil incubation experiments, increasing N mineralization rates were found up to this pH level (Fu et al., 1987; Curtin et al., 1998). Most probably the lower NH_4_^+^ accumulation in the organically fertilized peat at pH 6.5 was due to higher NH_3_ emissions compared to pH 5.5 (**Figure 3C**). Based on the aerial NH_3_ concentrations detected above the substrate surface, the gaseous N losses were most intensive within the first week of cultivation. Accordingly, in field experiments with urea application, NH_3_ volatilization was up to one order of magnitude higher during the first days after fertilization compared to the following period. In total, more than 60% of the applied urea can be lost *via* NH_3_ (Black et al., 1985; Pacholski et al., 2006).

The open-top chamber approach used in this study is not suitable for quantifying absolute NH_3_ volatilization. Nevertheless, significant NH_3_ losses from the growing medium can be assumed when taking into account the amounts of NH_4_^+^ detected within the first 2 weeks of cultivation. At this time of cultivation, uptake of mineral nutrients by plants is still negligible. On average not more than 60% and 40% of the applied AFF-N were detected as NH_4_^+^-N in the peat at initial substrate pHs of 5.5 and 6.5, respectively. Since amino acids are decomposed by soil microorganisms with a half-life of 1–12 h (Jones, 1999), most of the NH_4_^+^ release is to be expected within a few days. However, a certain part of the amino acids is retained in the microbial biomass (Barak et al., 1990). Beside this N immobilization, primarily NH_3_ emissions had probably contributed to the balance gap of applied AFF-N, especially at the higher substrate pH. Gaseous N losses by denitrification are usually relatively low in peat-based substrates if waterlogging or compaction is avoided (Agner and Schenk, 2005), as ensured in the trials presented here.

The increase in substrate pH immediately after organic base dressing reflects H^+^ consumption by ammonification (Ferguson et al., 1984). However, these temporary pH shifts were less pronounced with a higher initial substrate pH (**Figure 3B**). Firstly, this might be due to the logarithmic pH scale. The higher the pH, the more hydroxide ions (OH^-^) are required to increase the pH for one unit. On the other hand, with rising pH, NH_3_ is increasingly lost from the substrate. Each mole of emitted NH_3_ will increase the concentration of H^+^ by one mole (Sommer et al., 2004).

Ammonia concentration in the aerial environment of basil seedlings reached a maximum level of 1.8 ppm seven days after sowing (**Figures 3C** and **4C**). In the following weeks NH_3_ exposure decreased faster than expected compared to the relatively slow decline of NH_4_^+^ concentration and pH in the substrate (**Figures 3A, B** and **4A**, **B**). Noticeable changes in climatic conditions (e.g. air temperature, wind speed) can be excluded as possible causes. In field experiments, it was shown that the topmost millimeters of soils are most important for the volatilization of NH_3_ (Pacholski et al., 2006). Therefore, we assume that the NH_4_^+^ concentration and pH in the upper substrate zone dropped faster compared to conditions in the whole substrate.

In pure peat, nitrification was accelerated and increased by a substrate pH close to neutrality compared to more acid conditions (**Figures 3B, D** and **4B**, **D**). Similar results were reported by Lang and Elliott (1991) who identified a slightly alkaline pH as optimal for nitrification in peat-based growing media. At pH ≤ 5.4, nitrification was strongly inhibited. On the other hand, alkaline conditions can lead to an accumulation of NO_2_^-^ due to the inhibitory effect of high NH_3_ concentrations on *Nitrobacter* sp., which convert NO_2_^-^ to NO_3_^-^ (Bunt, 1988; Vetanovetz and Peterson, 1990 ). Accordingly, we observed a noticeable NO_2_^-^ accumulation in treatments with a substrate pH ≥ 7.0 during the onset of nitrification (**Figures 3E**, **4E**). However, if peat was amended with 5% (v/v) mature green waste compost and afterwards stored for 12 days before use NO_2_^-^ accumulation was suppressed (treatment 8). In this substrate mix pH was always between 6.0 and 7.0 and therefore in a range to prevent both inhibition of nitrification and accumulation of NO_2_^-^ due to low and high pH levels, respectively. Already 2 weeks after sowing two thirds of the mineral N in the peat-compost blend were converted to NO_3_^-^. In contrast, in a peat substrate without compost amendment a similar proportion of NO_3_^-^ was reached about 5 weeks later (**Figures 4A, D**). Mature composts usually contain high numbers of nitrifying bacteria (Chroni et al., 2009; Zeng et al., 2012) and thus can serve as an inoculum to enrich peat with these microorganisms (Delics et al., 2017). Nevertheless, apparently it took a couple of days before the nitrifying community was fully established in their new environment. This was indicated by results obtained with a peat-compost blend that was stored just 1 day before use (treatment 9). As a result, NO_3_^-^ accumulation lagged about 2 weeks behind compared to the same substrate mix that was previously stored for 12 days.

With ongoing nitrification, substrate pH decreased slightly (**Figures 3B**, **4B**). This pattern reflects the generation of 2 H^+^ ions during the microbial oxidation of NH_4_^+^ to NO_2_^-^ (Sahrawat, 2008). In pure peat, the pH decline continued until the end of the trial. In the later course of cultivation, plants might also contribute to the weak acidification of the growing medium since the uptake of NH_4_^+^ by roots is accompanied by an equivalent H^+^ efflux (Schubert and Yan, 1997). In peat-compost blends substrate pH turned once again during the last 2–3 weeks. Most probably the slight pH increase in this phase was triggered by the repeated AAF top dressings as well as the H^+^-consuming NO_3_^-^ uptake of plants.

### Impact of NH_3_, NH_4_^+^, and NO_2_^-^ Exposure on Basil Growth

In the experiments, basil was exposed to different concentrations of NH_3_ and NH_4_^+^ right from the beginning of cultivation. This was done by varying AAF base dressing rates in combination with different initial substrate pH values. The emergence of radicle and germ bud from seed was not affected by any of the treatments examined. Obviously, the presence of ammonical N did not interfere with physiological processes involved with the onset of basil germination. However, shortly afterwards seedlings were suffering from increased NH_3_ and NH_4_^+^ exposure. Under these conditions, the development of radicle, hypocotyl, and cotyledons was strongly inhibited (**Figure 5B**). Similar adverse effects of ammonical N forms on germination and seedling development were reported for several plant species (Bremner and Krogmeier, 1989; Ells et al., 1991; Britto and Kronzucker, 2002; Qi et al., 2012; Bergstrand et al., 2018).

The number of surviving seedlings and their following growth performance were negatively correlated with the intensity of NH_3_ and NH_4_^+^ exposure occurring 7–21 days after sowing (**Tables 2**, **4** **,** and **5**). Overall, these relationships were stronger for NH_3_ and more pronounced between November to April (experiments 2 and 3) than in September/October (experiment 1). Thus, it seems that growing conditions prevailing in the winter months increased the ammonical susceptibility of basil. On the other hand, it is also conceivable that NH_3_ emitted from the substrate remained for a longer time in the plant canopy due to the restricted ventilation of greenhouses in the colder season. Accordingly, it is known from commercial organic basil production that growth impairments are more severe in the winter cultivation period. Besides stunted plant growth, typical symptoms are chlorotic and necrotic cotyledons, frequently accompanied by fungal diseases such as *Botrytis* (Frerichs et al., 2017). Surprisingly, in this study cotyledons always remained green. This was contrary to previous findings even with the same basil cultivar under similar cultivation conditions (Frerichs et al., 2017). Thus, high exposure to ammonical N does not necessarily involve cotyledon discoloration.

Critical levels of ammonical N exposure in the early development stage of basil (7–21 days after sowing) were reached at concentrations of 0.1-0.2 ppm NH_3_ in the aerial environment of plants and 50–100 mg NH_4_^+^-N L^-1^ in the growing medium. At higher concentration levels, the number of plants, fresh matter yield, and plant height were diminished by more than 10% (**Table 6**). With regard to NH_3_, basil seems to be more sensitive than many other food crops, as reviewed by Krupa (2003). However, most of the published data related to this aspect were based on exposure experiments with plants in the post-emergence stage. A study on wheat has shown that the seed germination of this cereal species is unaffected at NH_3_ concentration below 0.3 ppm, but completely inhibited at 0.8 ppm (Pairintra, 1973). For pot grown basil, a poor germination and weak plant growth was observed when the NH_4_^+^-N concentration was between 100–130 mg (L substrate)^-1^ in the first 3 weeks after sowing (Bergstrand et al., 2018). In contrast, Frerichs et al. (2017) reported that the germination process of basil was not adversely affected by NH_4_^+^-N levels at about 200 mg (L substrate)^-1^. In this experiment, the substrate pH was slightly acidic throughout the entire germination period and thus, NH_3_ can be assumed to be negligible. Under these circumstances, basil seemed to tolerate up to 300 mg NH_4_^+^-N (L substrate)^-1^ (**Table 6**). However, after germination is completed and seedlings start to take up nutrients from the growing medium much lower NH_4_^+^-N concentrations should be present to ensure proper plant growth.

High NH_4_^+^ and NH_3_ concentrations in the early plant development stage had a long-lasting adverse impact on the growth of basil. This became evident by the fact that plants in treatment 9 generated 41% less shoot biomass than plants in treatment 8 (**Table 5**), although both were grown under moderate to low ammonical exposure in the second half of cultivation period. However, seedlings in treatment 9 were subjected to distinctly higher concentration levels of ammonical N, especially of NH_3_ (**Figure 4**).

In the organically fertilized treatments, NO_2_^-^ concentrations reached a maximum level of 42 mg (L substrate)^-1^ (**Figures 3E**, **4E**). Harmful effects to plants can be expected if NO_2_^-^ concentration exceeds 5 mg L^-1^ in the root zone (Zsoldos et al., 1993; Hoque et al., 2007), especially in the seedling stage (Bergmann, 1993). However, NO_2_^-^ appeared only temporarily in a few pots. Thus, NO_2_^-^ concentration was mostly still very low in the second week of cultivation when growth impairments on seedlings became visible. Hence it seems unlikely that NO_2_^-^ significantly contributed to these adverse effects. Nevertheless, further investigations are needed to examine the accumulation of NO_2_^-^ in growing media after organic fertilization and to clarify the sensitivity of basil against this inorganic N species.

### Impact of pH and Other Substrate Parameters on Basil Growth

Plant height and fresh matter yield of basil were inversely related to the substrate pH in experiments 2 and 3 (**Tables 2**, **4** **,** and **5**). Besides the increasing NH_3_ exposure, other factors might have limited the crop development at neutral to slightly alkaline conditions. This was particularly noticeable in experiment 3. Plants predominantly fed with NO_3_^-^ (treatment 6), were not exposed to detectable amounts of NH_3_ but showed a stunted growth. Probably this was mainly due to a low availability of P in the growing medium (**Figure 4F**). According to Meinken (2008), a concentration ≥ 22 mg P (L substrate)^-1^ extracted by CAT is required to supply horticultural crops having a high nutrient demand sufficiently with P. This level was not reached in the pure peat substrates within the first 4 weeks of cultivation. As a result, typical P deficiency symptoms such as violet discolored cotyledons emerged. Subsequently, P supply in the substrate was increased to the target range by means of top dressing with a water-soluble P fertilizer. Simultaneously, P availability was presumably improved by the declining substrate pH (**Figure 4B**), which might have increased the solubility of apatitic compounds (Alt et al., 1994).

Despite sufficient P supply in the second half of cultivation, growth of plants in the control treatment of experiment 3 remained distinctly lower than usually observed for NO_3_^-^ fertilized basil. In both previous experiments NO_3_^-^-fed plants generated the highest fresh matter yield (**Table 2**). On the one hand the better crop performance might be related to the generally lower substrate pH in these trials (**Figure 3B**). On the other hand, the characteristics of the different apatitic P fertilizer types could have played a role. In experiment 3 a bone meal based fertilizer was used. In principle, this product exhibits a higher P solubility at increased pH than rock phosphate (Möller, 2015), which was applied in experiments 1 and 2. However, the bone meal fertilizer consisted of coarser particles (80% < 2.0 mm) than the ultrafine-sieved rock phosphate (80% < 0.1 mm). Since the percentage of dissolution of apatitic P strongly increases with decreasing particle size (Kanabo and Gilkes, 1988), it is assumed that the bone meal fertilizer was less effective at supplying phytoavailable P.

Basil growing in compost-amended peat did not show any P deficiency symptoms, although the initial P supply was equally adjusted in all treatments. Nevertheless, in pure peat substrates lower CAT-extractable P concentrations were observed at the beginning of the cultivation period. It is most likely that the positive effect of the compost on P availability was a result of the faster pH decline in this substrate mix (**Figures 4B, F**).

Iron absorption of basil was also hampered at increased substrate pH, as indicated by a yellowish discoloration of the primary leaves. These deficiency symptoms were mainly visible in the second experiment in pure peat (**Figure 6E**). At neutral to slightly alkaline conditions Fe ions react rapidly with oxygen and OH^-^ ions to form barely soluble compounds (Lindsay and Schwab, 1982). These precipitation processes may occur even if the fertilized Fe is chelated by ethylenediaminetetraacetic acid (EDTA) or N-(2-hydroxyethyl)ethylenediaminetriacetic acid trisodium salt hydrate (HEDTA). To alleviate arising Fe deficiency symptoms quickly, foliar sprays with Fe chelates are an effective tool (Fisher et al., 2003), as applied by an overhead irrigation in experiment 3.

A stabilization of the substrate pH against alkalization as well as acidification shifts can be attained by using peat substitutes with a high buffering capacity, such as composted bark (Neumaier and Meinken, 2015).

### Approaches to Mitigate Harmful NH_3_ and NH_4_^+^ Effects

To improve the growth of organically fertilized basil, strategies are needed which contribute towards less intense and shorter NH_3_ and NH_4_^+^ exposure of plants. In this respect, first of all, it seems reasonable to supply moderate organic N base dressing rates [≤ 250 mg N (L substrate)^-1^] and to adjust the substrate pH to about 5.5. When basil is sown in a peat-based growing media with these characteristics, the exposure of seedlings to ammonical N will remain below or close to the above-mentioned critical concentration levels (**Figures 3A, C** and **Table 6**). To meet total N requirements of basil, base dressing can be supplemented by repeated top dressings in the later course of cultivation (Koller et al., 2014), without leading to harmful ammonical N concentrations. Nevertheless, even by using these measures, a retarded plant growth compared to mineral N-fertilized basil can be expected (**Table 2**). This results most probably from the high NH_4_^+^-N/NO_3_^-^-N ratio in the substrate remaining for several weeks after organic base dressing. A previous study indicated that biomass production of pot-grown basil was highest at balanced to NO_3_^-^-dominated N nutrition (Frerichs et al., 2019). In pure peat substrates it usually takes more than 4 weeks before NO_3_^-^-N concentration reaches a similar level to NH_4_^+^-N. The amendment of peat with mature green waste compost has proven to be a suitable method to accelerate the NO_3_^-^ formation in the substrate. The best results will be obtained when the compost-peat blend is stored for several days before use. In this way organically fertilized basil will be fed already from the seedlings stage onwards with adequate amounts of NO_3_^-^.

In the present experiments, a compost amendment of 5% (v/v) to the peat was sufficient to realize the outlined positive effects. In commercial organic basil cultivation even higher compost proportions are chosen and often required by organic farming associations as well. This may possibly boost the impact of the compost. However, Delics et al. (2017) found a nearly similar nitrification pattern in an unstored peat-based substrate that contained 30% (v/v) green waste compost to the one that we observed by using just one sixth of this admixture with a short-term storage of the substrate blend for 1 day. Nevertheless, further investigations are necessary to assess the relevance of the compost/peat blending ratio for the intended purpose. Furthermore, the influence of the duration and conditions of storage (e.g., water content of the substrate mix, aeration, storage temperature) on the N dynamic in compost-peat mixtures have to be examined in more detail.

For a successful implementation of the proposed methods it is certainly essential to use fully mature compost. The maturity level of composts can be easily recognized when the temperature of the organic material approaches the ambient range and the NH_4_^+^-N/NO_3_^-^-N ratio falls below 3 (Cáceres et al., 2018).

## Conclusions

The exposure of seedlings to ammonical N was found to be a cause of growth impairment frequently observed in organic basil production. Critical concentrations were reached at 0.1-0.2 ppm NH_3_ in the aerial environment and 50–100 mg NH_4_^+^-N (L substrate)^-1^ in the root zone of plants. However, when NH_3_ is absent and sufficient amounts of NO_3_^-^ are available in the growing media, basil seems to tolerate higher levels of NH_4_^+^. Therefore, a fertilization strategy is recommended which combines a moderate organic N base dressing with repeated top dressings in the later course of cultivation to meet the total N requirement. Easily decomposable organic fertilizers such as those based on amino acids should be used to ensure a rapid N mineralization. By adjusting the initial substrate pH to 5.5–6.0 the formation of NH_3_ during the ammonification of organic compounds would be prevented. Furthermore, the addition of mature green waste compost to peat-based substrates can subsequently promote the nitrification of NH_4_^+^, most noticeably if this substrate blend is stored for several days before use. With this approach, it seems possible to supply basil a substantial proportion of NO_3_^-^ right from the seedling stage, and thus to improve crop growth performance. Further investigations are, however, needed to evaluate whether this procedure can be also successfully used in the organic production of other pot-grown crops.

## Data Availability Statement

The datasets generated for this study are available on request to the corresponding author.

## Author Contributions

CF, DD, and AP conceived and designed the experiments and wrote the manuscript. CF performed the experiments and analyzed the data.

## Conflict of Interest

AP was employed by EuroChem Agro GmbH during investigations and production of the manuscript.

The remaining authors declare that the research was conducted in the absence of any commercial or financial relationships that could be construed as a potential conflict of interest.

## Abbreviations

AAF, Amino acid fertilizer; BD, Base Dressing; CAT, CaCl_2_/DTPA; TD, Top Dressing.
